# Supplementary material for: Lessons learned to boost a bioinformatics knowledge base reusability, the Bgee experience
Source: Gigascience. 2023 Aug 17;12:giad058. doi: 10.1093/gigascience/giad058 (PMC10433096; doi:10.1093/gigascience/giad058)
Supplement: giad058_Response_to_Reviewer_Comments_Original_Submission [file giad058_response_to_reviewer_comments_original_submission.pdf]

Author's Response To Reviewer Comments

Close

Dear Editors and Reviewers,

We would like to thank the reviewers for all their insightful comments, improvements and suggestions and the editors for considering our article for a possible publication in GigaScience.

We have revised the paper in order to take all comments into account. We thus hope that this new version of our article complies with the remarks and answers the questions raised by the reviewers.

The following paragraphs list every issue identified by the reviewers, along with our related answers. Finally, to facilitate the review process, the modified and added sentences are highlighted in yellow in the revised version of our article.

Reviewer 1: “I agree with their position on "FAIR principles", which are intrinsically related, should not be dissociated and definitely need more practical evidences (as they were in principle under specified); I did contribute to this topic myself with a paper just accepted at the CAiSE conference 2023.”
Authors: Thank you very much for your interest and to point out this paper. We are now citing the referred paper in our article.

Reviewer 1:- The paper seems more a discussion on the potential of Bgee, at times a documentation addendum to that system, rather than a proper dissertation on the problem of improving interoperability/reusability in scientific data knowledge bases.
Authors: We hope that by considering the reviewers’ suggestions/corrections the revised version of the paper addresses this issue too.

Reviewer 1: “1) According to this impression, I am questioning the suitability of the chosen title. In my opinion, Bgee should be included in the title, which otherwise suggests a broader discussion on the topic.”
Authors: We changed the title to better correspond to the paper contents by also explicitly including “Bgee” in the title.

Reviewer 1: The last portion of the introduction makes an attempt to justify why the paper is focused only on Bgee and why its teachings can be extended to other KBs. It makes this point by citing [7] and [15]. I do not agree with this statement, unless it was much more motivated. At least 3 or 4 examples of KBs should be analyzed in depth to be able to make a general discussion on the topic. Or, at least, a comprehensive comparison / parallel should me made with other KBs in order to justify how results can be 'transferred' from Bgee to them.
Authors: We are now justifying our work is transferable to other KBs by highlighting similar implementations, when it is applicable, by three major bioinformatics KBs, notably, GeneCards (a human gene-centric KB), UniProtKB (a protein-centric KB) and Orthologous MAtrix (OMA, an orthology resource). For the later one, we also wrote a dedicated subsection to demonstrate that thanks to one of the one-side interoperability approaches available by OMA, Bgee is able to consume its data, that is, to interoperate with it. Therefore, from the point of view of the data consumer rather than the data producer to be able to make a general discussion on the topic.

Reviewer 1: Else, the title and introduction should be frank in presenting the Bgee-centric results.
Authors: We changed the title and introduction to reflect the results are based on the Bgee team experience.

2) I also find the title somewhat incoherent w.r.t. to the introduction message: "we will describe how to

improve reusability through interoperability enhancement", whereas "boosting interoperability" seems to be the goal in the title.
Authors: Thanks for the remark. We replaced “interoperability” with “reusability”.

Moreover, the choice of focusing only on interoperability should be explain more thoroughly. I.e., it is not sufficient to say: "We focus on the interoperability aspect because without it, for instance, it will be hard or even impossible to be findable and accessible."
Authors: We added a few sentences to reinforce why we are focusing on interoperability as follows: “Moreover, achieving interoperability has been well recognised as a complex task by several researchers [1,2,3,4]. Since it is a hard task, it also presents an impediment to the exchange of information among independent bioinformatics KBs. Therefore, to mitigate this issue, in this paper, we focus on pragmatic approaches for interoperability enhancement of bioinformatics KBs…”
[1] https://www.doi.org/10.5555/2962664.2962674
[2] https://www.doi.org/10.1007/978-3-030-56883-2_2
[3] https://www.doi.org/10.1007/978-3-319-20765-0_7
[4]10.1109/BigData.2014.7004486

- The paper sounds more like an 'experience paper' rather than a research paper. This is also corroborated by the high number of provided self-citations. If this is the intent of the authors, I suggest it is made more explicit in the introduction (which would require discussing other KB-solutions, existing prior to or in parallel with Bgee) and also in the methods: here, a reader does not perceive the exploratory vibe (which would require discussing methodological alternatives and why those used in Bgee were preferred).
Authors: We changed the article title and made it more explicit in the introduction that we report our experience. We also added various discussions about other KB-solutions for each Bgee experience in Section 4 to further demonstrate the lessons learned are applicable and transferable to other bioinformatics KBs. Moreover, we agree with the reviewer's comments about the article type and we would like to kindly highlight we changed during submission (before peer-review) the paper category from “a research article” to “a review article” category as suggested by the GigaScience’s editor as being the most suitable category in the GigaScience journal for an experience-type paper like ours.

- The paper needs an improvement in the pragmatics and rationalization. Section 3 is organized as a report of things that have been done. A research paper on using interoperability to achieve reusability deserves a more organized discussion. More specifically: the 'use cases' in section 3 read as the description of source-specific modules that the developers of Bgee had to design (even collaborating with stakeholders of other sources, e.g., GeneCards) and implement in order to allow data exchange between Bgee and those specific systems. This problem could be overcome with at least: 1) An overall architecture schema that allows a general overview of the list of components that have been developed; 2) Proposing a general workflow (inspired to the one of Bgee) that can bee reused (or is already reused in other KBs cases, if this applies).
Authors: We find the reviewer’ suggestion highly valuable and we included in the paper an overall architecture schema. However, we would like to kindly highlight that before peer-review and at the submission moment, the article category changed from a research paper to a more appropriate category as suggested by the GigaScience editor, hence, it is not classified as a research article.

- The choice of having Definitions within what are called 'use cases' is quite unusual and deviates from the focus of the discussion. As these concepts seem to be novel, it would be better to dedicate them more space and possibly a visual schema. A taxonomy of interoperability approaches, according to the experience of the authors would improve the value of the paper. I would move the definitions 1, 2 and 3 outside of the use cases, making them a methodological distinction, by means of which the cases are categorized. Additional note: the authors speak about 'federation of knowledge bases' (e.g., in page 8, speaking about Bgee and OncoMX). I wonder if this terminology corresponds to one of the three definitions above or is yet another kind of interoperability pattern.
Authors: Thanks for the suggestions. We created a dedicated section (Section 3) for the definitions along with more explanations and a table as suggested by the other reviewer that includes classifications and summarizes each use case.

Some additional discussions could be interesting:

- Related to Lesson 9, a discussion could be added on those KB sources that - instead - necessarily need to be not public (or that make a value out of it), requiring a strict policy of access to their data, with the intention of protecting the data ownership of its authors, but in fact hindering many possibilities for interoperability and open science (e.g., GISAID for viral genomics).
Authors: Thanks! We included your suggestion.

- I wonder if some parts the paper refer to 'reusability' concept as it was just 'usability' (e.g., in Lesson 1)
Authors: This highly depends on the context. Because of this, we provided a definition of reusability in the first paragraph of our article: “the capability of a resource to be used multiple times by distinct agents”. Therefore, the fact of being reusable is related to the capacity of being potentially useful for different/numerous users and tools. Moreover, to improve understandability we replace in Lesson 1 “reusability” with “(re)usability”, since both terms are applicable in this lesson.

- Delegates of KBs and their interoperability are often involved in the text (either explicitly or by calling 'them'). This actor in the process should be further detailed, providing a more precise description of the context in which interoperability is targeted/achieved.
Authors: Thanks for suggesting this additional discussion that could be interesting. We thoroughly tried to consider this suggestion, however we are afraid that it may deviate the discussions out of the main scope of the paper, that is the generalization of the lessons learned to be applicable by other knowledge bases that do not necessarily have the same kind of actors involved.

Some minor considerations:

- the paper makes claims about bioinformatic knowledge bases, but uses as a single use case Bgee, which is claimed to be a database (by the same group of authors). Please clarify this mismatch, which seems to me not only terminological.
Authors: Thanks to pointing this out, Bgee is indeed a knowledge base and we replaced the term database in our article with knowledge base. Unfortunately, both terms are often interchangeably used in bioinformatics.

- I do not particularly agree with the choice of merging structural and semantic-level heterogeneity, considering schema as semantic (page 3, beginning of section 2), could this be further supported?
Authors: We decided to remove the statement: “we consider structural heterogeneity as a semantic heterogeneity”. We agree that can lead to controversial discussions. Our initial intention was to simplify the understanding to a broader audience (i.e., GigaScience readers) but it should not compromise the information accuracy.

- The paper contains too many points where the reader is referred to forward parts of the manuscript, leaving concepts unclear.
Authors: Thanks for this remark. We removed several of them without compromising the understanding, and in doing so, we also improved readability.

- Fig 2 does not add much information, What should a reader understand from it? This could be added to the caption, if deemed important by authors.
Authors: The figure was removed.

- Some statements are not really justified by the provided examples "Therefore, to achieve interoperability among different KBs, solving syntactic and semantic heterogeneties is the key to success." and the feeling, as a reader, is that the narrative does not have good argumentations.
Authors: Thanks for this feedback. We have removed the statement to avoid the reported feeling without compromising the article readability.

- Some statements result quite obvious (e.g., 'the lack of data structure and semantic representations generate ambiguities and misinterpretations, and consequently, heterogeneities that may result in non-compliant files for the NCBI LinkOut file reader by the data producer') while not really backed by insightful analyses.
Authors: Thanks for this remark. We have rephrased the statement. We would also like to reinforce some

statements even if they seem obvious, we tried to be careful in order to improve the understanding by a broader audience among GigaScience readers. This is because they are from diverse research areas in life and biomedical sciences.

Please correct some typos:...
Authors: Thank you very much for the careful reading. All reported typos were corrected.

Reviewer #2: The paper "Boosting interoperability: towards an increasingly reusable bioinformatics knowledge base" provides detailed information about use cases implemented by the Bgee to enhance its interoperability with other databases. The use cases described are important as they provide knowledge to the community and help other interested parties find solutions to similar challenges. The paper is well-written, falls within the scope of the journal, and makes a valuable contribution to the literature. However, certain aspects of the paper could benefit from greater clarification.
Authors: Thanks, we hope with the revised version of the manuscript we addressed all reviewers' concerns.

In the introduction, it is mentioned that ontologies only "elevate heterogeneity problems at a higher level". Although I agree with this statement, I understand that the use of ontologies can also result in fewer heterogeneity problems (i.e., heterogeneity occurrences). Specifically, I argue that the number of ontologies that define overlapping domains or concepts is significantly smaller than the number of datasets that collects information about the same domains or concepts. I think the authors share the same thought, but the way these observations are presented in the paper creates room for different expectations. When I first read the sentence above, I expected that the authors would discuss possible solutions to the problem of ontology heterogeneity throughout the paper, which did not happen.
Authors: Thanks for spotting this. In principle, our idea was to provide broad concepts and issues when implementing and performing interoperability. Nevertheless, we understand that this can create room for different expectations. Because of this, we have simplified the paragraph that mentions the highlighted problem without compromising the readability.

I like the distinction between different types of interoperability (one-side, two-side and multi-side). However, I feel that the relationship between the types and the use cases is not clearly presented, and I have some difficulty understanding which type of interoperability approach is used in each case. I suggest including a summary table with the cases, and the interoperability approaches used in each one (and maybe other important points?).
Authors: Thanks for the suggestion. We added a table in Section 3 summarizing them.

About one-side and multi-side interoperability: I wonder how to classify a case where one side strictly complies with the other side (one-sided?), but the other side implements a third-party standard (multi-sided?). The true question behind this is: are the definitions of interoperability types clear enough? It seems to me that there are cases in which more than one classification is possible.
Authors: Thanks for pointing this out. To clarify this, we have reviewed the definition of one-side interoperability and provided further explanations. For clarity, we moved the definitions to a new Section (Section 3). Concerning your example, we classify it as a multi-side interoperability, because, in the end, both sides must comply with a third-party standard since one side has to comply with the multi-side interoperability implemented by the target system.

Finally, I have a few questions regarding the use cases. In general, it is unclear to me why a particular approach is used in each case, whether other approaches are possible, and what the pros and cons are. For example, why exchanging CSV files in some approaches and implementing a federated query in others? Are these choices made because they are the only viable option? Disclosing how the authors came up with these recommendations would help readers to identify the best approach for their use cases. Overall, the paper would benefit from adding a discussion section that contrasts and compares the use cases.
Authors: Thanks for reporting this issue. We added a subsection called “Final implementation considerations” just before the 10 lessons learned section to address the reported problem.

Close
