# Supplementary material for: Lessons learned to boost a bioinformatics knowledge base reusability, the Bgee experience
Source: Gigascience. 2023 Aug 17;12:giad058. doi: 10.1093/gigascience/giad058 (PMC10433096; doi:10.1093/gigascience/giad058)
Supplement: giad058_Response_to_Reviewer_Comments_Revision_1 [file giad058_response_to_reviewer_comments_revision_1.pdf]

Author's Response To Reviewer Comments

Close

Dear Dr. Nicole Nogoy (editor) and reviewers,

We thank the editor for the opportunity to resubmit our article for publication in GigaScience. We have revised the paper again in response to the additional comments of Reviewer 2. We thus hope that this version of our article complies with all comments and answers the questions raised by the reviewer and the editor. We would also like to thank Reviewer 1 for accepting our paper after the first round of revisions. The following paragraphs list every comment by the Reviewers, along with our related answers and rebuttals.

Reviewer 1: Typo: Page 11, right column, last paragraph; make sure that the letter C is typed similarly to A and B.
Authors: Thanks, the font was corrected.

Reviewer 2:

I agree with reviewer #1 that the first version of the paper seemed more like a discussion of the potential of Bgee. Although the new proposed title and the additions to the main text partially address this issue, I am not convinced by the changes in the abstract. When the authors say "The findings are driven by several real-world scenario examples that were mostly implemented by Bgee, a well-established gene expression database, and other major knowledge bases", it still gives the same impression as the first version of the paper. Overall, I feel that the abstract has not kept up with the improvements in the rest of the paper. For example, I suggest that the abstract should say something similar to the last paragraph of the introduction: "To further demonstrate they are transferable, for each Bgee interoperability experience, we will also highlight, when it is applicable, similar implementations by widely used bioinformatics KBs such as GeneCards (a human gene-centric KB), UniProtKB (a protein-centric KB) and Orthologous MAtrix (OMA, an orthology resource)."
Authors: We included your suggestion in the abstract. The few words we added in the abstract after the first revision (i.e, "and other major knowledge bases") were to summarise this idea, but we recognize it is not necessarily clear from just reading the abstract.

In the highlighted reusability sentence in the first paragraph of the introduction: are you suggesting a new definition of reusability for your own work or reusing one from literature? If the latter, please include a reference (NB: the definition of interoperability is referenced).
Authors: We added a sentence at the end of the first paragraph to make it clearer. As a reminder, this reusability definition was already stated in the first version of the paper but between parentheses in the paragraph. We decided to move out of parenthesis to improve clarity. The definition is a generalisation of the following one extracted from [1]: "Data reusability is the capability of data to be used (several times) by different programs concurrently, or at different times". Because, we extended the definition to be applied not only for data but any resource that can be used by either a computer program or a user (human), that is an agent. Finally, we would like to note that the definition in [1] precedes the FAIR principles by more than two decades.
[1] https://dl.acm.org/doi/10.1145/130069.130134.

I also wonder about the claims made in the second half of the second paragraph of the Introduction. I partially disagree that the principles should not be dissociated nor seen as independent. A FAIRification project should indeed have findability, accessibility, interoperability and reusability as its ultimate goal. However, when the letters are broken down into the 15 principles, the dependency claim no longer holds. This is because some principles are more closely related to some than others, and because some should be prioritised differently depending on the FAIRification objectives. For example, A1.1 and A1.2 are more

dependent on each other than on F1. Also, some principles (e.g., F2) can be achieved in different manners depending on the domain where they are being applied. Finally, there must be room for some independence in order for the FAIR implementation to be viable (it is infeasible to implement all the principles at once).
Authors: we believe that further discussions about the FAIR principles are out of scope of this article, as it is not a review about the FAIR principles themselves. Furthermore, we are not stating that the FAIR principles `must not` be dissociated, our assertion is that they `ideally should not` “Ideally” is an important keyword in our statement , i.e., “the FAIR principles are intrinsically related and that, ideally, they should not be dissociated nor seen as independent” extracted from our article. The aim of our paper is to help and contribute to improve reusability of bioinformatics knowledge bases. In addition, we would like to kindly ask the editor to mediate this comment that seems contradictory between reviewers. As a reminder, Reviewer 1 during the first revision stated the following: “I agree with their position on FAIR principles, which are intrinsically related, should not be dissociated and definitely need more practical evidences (as they were in principle under specified); I did contribute to this topic myself with a paper just accepted at the CAiSE conference 2023.”. Finally, the contents of this paragraph has not been modified since the submission of the first version of the paper and it is odd that Reviewer 2 makes this comment at this stage.

Secondly, I do not think that publications 3 and 4 lead to the conclusion that the structure and abstraction of the principles in four different letters has been the source of confusion and misinterpretation by researchers. Some may get the impression that these claims come from such publications (e.g. as a motivation for writing these papers), which is not true. I acknowledge the problem of confusion, but the authors also fail to mention that these principles have been made "abstract" (I would say agnostic) to respect the particularities of each research community. That is, the principles do not dictate an exact implementation, as this should be defined by each community according to its own needs.
Authors: Unfortunately, we think the reviewer 2 misunderstood this paragraph during the second round of revision. We would like to point out this paragraph part was not modified since the first submission. To be clear, we are not citing 3 and 4 as publications leading to any conclusion, but to highlight our opinion that later publications were needed in order to better interpret the FAIR principles. That being said, it is not because the principles are “abstract” (or agnostic) they should not be clear. More specifically, the citations refer to this sentence extracted from our article:
“when the original authors needed to explain their initial intent and interpretations of the FAIR principles in later publications [3, 4]”.
We would like to invite the editor and the reviewer to read the following extracted sentences from [3] and [4] that support our statement.
“Before presenting an interpretation of the FAIR principles, it is useful to provide context around some of the concepts used in the formulation of the guiding principles that seem to have generated confusion in the early adopter community.” and we also recommend to read the whole section 2 in [3];
“As support for the Principles has spread, so has the breadth of these interpretations. In observing this creeping spread of interpretation, several of the original authors felt it was now appropriate to revisit the Principles, to clarify both what FAIRness is, and is not.” [4]
[3] https://direct.mit.edu/dint/article/2/1-2/10/10017/FAIR-Principles-Interpretations-and-Implementation
[4] https://content.iospress.com/articles/information-services-and-use/isu824

I have also had second thoughts about the example given in the third paragraph of the introduction. I think it is possible to be findable or accessible without being interoperable (in fact, this assertion contradicts the authors' claim that the four letters cause confusion). The authors seem to be collapsing different technical/semantic layers to which FAIR can be related. In other words, there are different levels of interoperability to be addressed, e.g. technical (the example given is the authors) and semantic (on the data and metadata). A counterexample would be a data resource that is findable through its metadata (because the metadata could be interoperated/managed by different systems (e.g. the server and the indexer/search engine)). However, the data that the metadata points to is not interoperable at all. I understand that this statement is based on the authors' own interpretation of interoperability (as given at the beginning of the same paragraph). This claim
should be accompanied by a statement that "F" and "A" are not possible without "I", according to the authors' understanding of Interoperability. Alternatively, a more appropriate fix would be to change the

motivation (e.g., "I" is one of the most targeted aspects of FAIR).
Authors: in our article, we are not redefining what is interoperability or having second interpretations or thoughts. We are strictly based on existing definitions of interoperability, more precisely we are quoting in our article the following definition of interoperability from an IEEE standard [1]: "the ability of two or more systems or elements to exchange information and to use the information that has been exchanged" [1,2,3]. It seems that Reviewer 2 might have misunderstood our statements during this second round of revision. We say that “we focus on the interoperability aspect because without it, for instance, it will be hard or even impossible to be findable and accessible”, which is different from stating that “without interoperability it is not possible to be findable and accessible” as interpreted by Reviewer 2. Reviewer 2 seems to have missed relevant keywords in our statements, e.g. “for instance, it will be hard ”, more precisely the fact that we do not say it is completely impossible but it can be “hard”. Finally, from our point of view, the provided counterexample is not coherent because despite the fact of “the data is not interoperable” (quoting Reviewer 2’s words), the moment we communicate, for example, the location of the data resource with some metadata (i.e., exchange of information), this means that data resource is only discoverable thanks to this exchange of information, that is interoperability.
[1] https://doi.org/10.1109/IEEESTD.2012.6228492
[2] https://doi.org/10.1016/j.compind.2013.09.001
[3] http://doi.org/10.1109/ICSTE.2010.5608843

The end of the last paragraph in the introduction section is slightly disconnected. In fact, this paragraph is very long and would benefit from some reorganisation. In the second part of the paragraph (i.e., "Furthermore, …"): are the authors citing related works? Are they also mentioned as similar implementations? I suggest elaborating this part as another paragraph focused on related works (or even considering making it a subsection).
Authors: Thanks for the suggestion. We divided the paragraph into several parts. After a more careful reading, we decided to remove the last part of the aforementioned paragraph that cites “experimental data tables”. Because, we believe these statements are out of scope of our work. This explains the disconnection mentioned.

I appreciate the improvements in clarifying the interoperability approaches and I think Table 1 has improved readability. However, I am not sure that Figure 1 provides any additional information compared to Table 1 and Figure 2. The technologies used are not linked to the knowledge base with which they were used, which makes the information a bit unclear. I suggest removing Figure 1 and adding the technologies used (e.g. RDF, Java API, Python API, etc.) as an extra column in Table 1 or making them all explicit in Figure 2.
Authors: The technical information requested is already described for each use case in Section 4 (the next section) and we think that including them in the table will be overwhelming. We are keeping Figure 1 because we would like to show highly relevant examples as a visual overview of what can be achieved by boosting reusability for non-technical readers. For example, the interoperability between a domain-specific scientific knowledge base (i.e., Bgee) and Wikipedia (a free encyclopedia).

Figure 2 is very informative and summarises the work well. I suggest adding some extra information/semantics to the relationships (the arrows). For instance, how should the arrow between "Generate Bgee data exchange files" and "EasyBgee SQL dump" be interpreted? Is it also an import? Or is it a "create" action?
Authors: We added an explanation in the legend to explicitly define the arrows' meanings.
Because, we think that adding more information directly in the Figure will visually overwhelm and reduce understandability due to the fact that most of them are self-explainable. For the aforementioned example, generating a SQL database dump file (e.g., “EasyBgee SQL dump”) is an export operation.

About Fig 11: I wonder how informative/complex it can be for the average reader. Should it be replaced by an infographic or a more didactic figure? Readers with a technical/related background could refer to a GitHub reference to the XML, which should be included in the text.
Authors: We included a persistent link to the referred XML file and a link to the Teiid documentation in the Figure’s legend that explains the semantics of the XML tags. As a reminder, the latter link was already provided in the “Bgee and OncoMX: a knowledge base federation” subsection.

For the sake of better organisation of the information/text, I suggest that "Final implementation considerations" becomes a subsection (i.e., 4.4).
Authors: We created the subsection as suggested.

Close
