# Supplementary material for: Lessons learned to boost a bioinformatics knowledge base reusability, the Bgee experience
Source: Gigascience. 2023 Aug 17;12:giad058. doi: 10.1093/gigascience/giad058 (PMC10433096; doi:10.1093/gigascience/giad058)
Supplement: giad058_Response_to_Reviewer_Comments_Revision_2 [file giad058_response_to_reviewer_comments_revision_2.pdf]

Author's Response To Reviewer Comments

Close

Dear Dr. Nicole Nogoy and Reviewer 2,

We thank the editor again for the opportunity to resubmit our article for publication in GigaScience. We have revised the paper in response to the additional comments of Reviewer 2. We thank the valuable time of Reviewer 2, that as reviewers ourselves, we strongly recognize the reviewers' efforts and valuable contributions to science.

We apologise to Reviewer 2 in case our previous responses were perceived as unfriendly. We decided to remove from our article all controversial statements related to the FAIR principles by considering the fact that this article is not a review about FAIR. As a result, we hope all raised comments are addressed. Please, find below our replies in more detail.

Reviewer 2:

I acknowledge that discussing FAIR is out of the scope of the paper. Nevertheless, the paper makes certain claims about the FAIR principles, and those must be addressed:
1. About my argument regarding the principles being seen as dissociated ones: I understand the authors' view on the (dis)association of the FAIR letters. My point was that F, A, I and R should not be seen as dissociated for resources aimed to be FAIR (e.g., a resource that is "F" and "R" is not considered "FAIR" - all letters are needed). However, while the letters should not be dissociated, the fifteen principles (e.g., F1, F2, A1..) and their implementation (here I mean implementation as a process) should be dealt with with certain independence. This is not contradictory to the comment made by Reviewer #1, but complementary. I invite the authors to revisit this section and check their claims one more time.
Authors: all controversial statements related to FAIR were removed from the introduction based on the fact that discussing "FAIR principles" is out of the scope of this paper. We also think that removing these statements contributes to improving the paper readability and to not deviate from the main focus of the paper, which is to improve bioinformatics knowledge base reusability.

2. In the prior round of reviews, I pointed out that references [3] and [4] do not support the claim that the four different letters have been the source of confusion and misinterpretation by researchers. The authors responded by stating that these references "highlight our opinion that later publications were needed in order to better understand the FAIR principles". I find it questionable to cite literature in order to make a stronger point about one's opinion. I suggest rewriting this section and make it clear that you are stating your own opinion.
Authors: this issue does not exist anymore because all controversial statements related to FAIR were removed from the introduction based on the fact that discussing "FAIR principles" is out of the scope of this paper.

3. Finally, let me come back to the example given in the third paragraph. I did not mean to say that the authors are having second interpretations or thoughts. I apologise if I conveyed this impression. Instead, I meant to say that your example makes sense according to the definition of interoperability used in this paper. However, this definition is not the only interoperability definition given in the literature. As mentioned in my previous response, there are different layers in which interoperability can be perceived, and there are different interoperability definitions for each layer. I suggest making it clear that the given example holds true in the context of the technical definition of interoperability that you are using. You could, for example, add an explanatory footnote.
Authors: the reported issue does not exist anymore. Because we removed the aforementioned example. Since all controversial statements related to FAIR have been removed from the introduction too. We did this based on the assumption that discussing "FAIR principles" is out of the scope of this paper.

Close
