# Supplementary material for: Lessons learned to boost a bioinformatics knowledge base reusability, the Bgee experience
Source: Gigascience. 2023 Aug 17;12:giad058. doi: 10.1093/gigascience/giad058 (PMC10433096; doi:10.1093/gigascience/giad058)
Supplement: giad058_Response_to_Reviewer_Comments_Revision_3 [file giad058_response_to_reviewer_comments_revision_3.pdf]

Author's Response To Reviewer Comments

Close

Dear editor,

All required files were uploaded. Note that there is no software application or general workflow to register for this work.

Please let us know if you need anything else from us.

Thank you very much for your support!

Best regards

Close
