# Supplementary material for: Lessons learned to boost a bioinformatics knowledge base reusability, the Bgee experience
Source: Gigascience. 2023 Aug 17;12:giad058. doi: 10.1093/gigascience/giad058 (PMC10433096; doi:10.1093/gigascience/giad058)
Supplement: giad058_Reviewer_1_Report_Original_Submission — Anna Bernasconi, Ph.D. -- 4/17/2023 Reviewed [file giad058_reviewer_1_report_original_submission.pdf]

Reviewer Report

Title: Lessons learned to boost a bioinformatics knowledge base reusability, the Bgee experience

Version: Original Submission **Date:** 4/17/2023

Reviewer name: Anna Bernasconi, Ph.D.

Reviewer Comments to Author:

I am glad to be involved in the process of review of this work. I am really interested in the topics discussed by the authors; authors demonstrate holding a deep understanding and wide experience in the practical implementation of a useful and widely-adopted system such as Bgee. In general, I agree with their position on "FAIR principles", which are intrinsically related, should not be dissociated and definitely need more practical evidences (as they were in principle under specified); I did contribute to this topic myself with a paper just accepted at the CAiSE conference 2023.
However, I have several concerns on the overall message and structure of this research paper. In the following, I try to make my concerns clear.
- The paper seems more a discussion on the potential of Bgee, at times a documentation addendum to that system, rather than a proper dissertation on the problem of improving interoperability/reusability in scientific data knowledge bases.
1) According to this impression, I am questioning the suitability of the chosen title. In my opinion, Bgee should be included in the title, which otherwise suggests a broader discussion on the topic. The last portion of the introduction makes an attempt to justify why the paper is focused only on Bgee and why its teachings can be extended to other KBs. It makes this point by citing [7] and [15]. I do not agree with this statement, unless it was much more motivated. At least 3 or 4 examples of KBs should be analyzed in depth to be able to make a general discussion on the topic. Or, at least, a comprehensive comparison / parallel should me made with other KBs in order to justify how results can be 'transferred' from Bgee to them. Else, the title and introduction should be frank in presenting the Bgee-centric results.
2) I also find the title somewhat incoherent w.r.t. to the introduction message: "we will describe how to improve reusability through interoperability enhancement", whereas "boosting interoperability" seems to be the goal in the title. Moreover, the choice of focusing only on interoperability should be explain more thoroughly. I.e., it is not sufficient to say: "We focus on the interoperability aspect because without it, for instance, it will be hard or even impossible to be findable and accessible."
- The paper sounds more like an 'experience paper' rather than a research paper. This is also corroborated by the high number of provided self-citations. If this is the intent of the authors, I suggest it is made more explicit in the introduction (which would require discussing other KB-solutions, existing prior to or in parallel with Bgee) and also in the methods: here, a reader does not perceive the exploratory vibe (which would require discussing methodological alternatives and why those used in Bgee were preferred).
- The paper needs an improvement in the pragmatics and rationalization. Section 3 is organized as a report of things that have been done. A research paper on using interoperability to achieve reusability deserves a more organized discussion. More specifically: the 'use cases' in section 3 read as the

description of source-specific modules that the developers of Bgee had to design (even collaborating with stakeholders of other sources, e.g., GeneCards) and implement in order to allow data exchange between Bgee and those specific systems. This problem could be overcome with at least: 1) An overall architecture schema that allows a general overview of the list of components that have been developed; 2) Proposing a general workflow (inspired to the one of Bgee) that can bee reused (or is already reused in other KBs cases, if this applies).
- The choice of having Definitions within what are called 'use cases' is quite unusual and deviates from the focus of the discussion. As these concepts seem to be novel, it would be better to dedicate them more space and possibly a visual schema. A taxonomy of interoperability approaches, according to the experience of the authors would improve the value of the paper. I would move the definitions 1, 2 and 3 outside of the use cases, making them a methodological distinction, by means of which the cases are categorized. Additional note: the authors speak about 'federation of knowledge bases' (e.g., in page 8, speaking about Bgee and OncoMX). I wonder if this terminology corresponds to one of the three definitions above or is yet another kind of interoperability pattern.
Some additional discussions could be interesting:
- Related to Lesson 9, a discussion could be added on those KB sources that - instead - necessarily need to be not public (or that make a value out of it), requiring a strict policy of access to their data, with the intention of protecting the data ownership of its authors, but in fact hindering many possibilities for interoperability and open science (e.g., GISAID for viral genomics).
- I wonder if some parts the paper refer to 'reusability' concept as it was just 'usability' (e.g., in Lesson 1)
- Delegates of KBs and their interoperability are often involved in the text (either explicitly or by calling 'them'). This actor in the process should be further detailed, providing a more precise description of the context in which interoperability is targeted/achieved.
Some minor considerations:
- the paper makes claims about bioinformatic knowledge bases, but uses as a single use case Bgee, which is claimed to be a database (by the same group of authors). Please clarify this mismatch, which seems to me not only terminological.
- I do not particularly agree with the choice of merging structural and semantic-level heterogeneity, considering schema as semantic (page 3, beginning of section 2), could this be further supported?
- The paper contains too many points where the reader is referred to forward parts of the manuscript, leaving concepts unclear.
- Fig 2 does not add much information, What should a reader understand from it? This could be added to the caption, if deemed important by authors.
- Some statements are not really justified by the provided examples "Therefore, to achieve interoperability among different KBs, solving syntactic and semantic heterogenetics is the key to success." and the feeling, as a reader, is that the narrative does not have good argumentations.
- Some statements result quite obvious (e.g., 'the lack of data structure and semantic representations generate ambiguities and misinterpretations, and consequently, heterogeneities that may result in non-compliant files for the NCBI LinkOut file reader by the data producer') while not really backed by insightful analyses.
Please correct some typos:
- reference 5 cannot be from 1970

- reference 74 is incomplete
- page 2, right column:
---5th row: there is no needs -> need
---24th row: imply -> implies
- page 3, right column:
---1st row: have -> has
---9th row (from bottom) 2 -> Figure 2
---9th row (from bottom) 3 -> Figure 3
- page 4, right column
---7th row: remove double ')'
---9th row: complies -> comply
- page 6, left column: the URL of sparql should be provided as a ref in the bibliography
- page 8, right column:
---lesson 1: is better -> it is better
---before 'Thus' a full stop is missing
- Lesson 2 could be rephrased as 'Iteratively improving the information exchange is better than trying to achieve full interoperability at once'
- remove the double full stop in Lessons 4-5 titles.

Methods

Are the methods appropriate to the aims of the study, are they well described, and are necessary controls included? Choose an item.

Conclusions

Are the conclusions adequately supported by the data shown? Choose an item.

Reporting Standards

Does the manuscript adhere to the journal's guidelines on [minimum standards of reporting?]() Choose an item.

Choose an item.

Statistics

Are you able to assess all statistics in the manuscript, including the appropriateness of statistical tests used? Choose an item.

Quality of Written English

Please indicate the quality of language in the manuscript: Choose an item.

Declaration of Competing Interests

Please complete a declaration of competing interests, considering the following questions:

- Have you in the past five years received reimbursements, fees, funding, or salary from an organisation that may in any way gain or lose financially from the publication of this manuscript, either now or in the future?
- Do you hold any stocks or shares in an organisation that may in any way gain or lose financially from the publication of this manuscript, either now or in the future?
- Do you hold or are you currently applying for any patents relating to the content of the manuscript?
- Have you received reimbursements, fees, funding, or salary from an organization that holds or has applied for patents relating to the content of the manuscript?
- Do you have any other financial competing interests?
- Do you have any non-financial competing interests in relation to this paper?

If you can answer no to all of the above, write 'I declare that I have no competing interests' below. If your reply is yes to any, please give details below.

I declare that I have no competing interests

I agree to the open peer review policy of the journal. I understand that my name will be included on my report to the authors and, if the manuscript is accepted for publication, my named report including any attachments I upload will be posted on the website along with the authors' responses. I agree for my report to be made available under an Open Access Creative Commons CC-BY license (http://creativecommons.org/licenses/by/4.0/). I understand that any comments which I do not wish to be included in my named report can be included as confidential comments to the editors, which will not be published.

Choose an item.

To further support our reviewers, we have joined with Publons, where you can gain additional credit to further highlight your hard work (see: https://publons.com/journal/530/gigascience). On publication of this paper, your review will be automatically added to Publons, you can then choose whether or not to claim your Publons credit. I understand this statement.

Yes Choose an item.
