# Supplementary material for: Lessons learned to boost a bioinformatics knowledge base reusability, the Bgee experience
Source: Gigascience. 2023 Aug 17;12:giad058. doi: 10.1093/gigascience/giad058 (PMC10433096; doi:10.1093/gigascience/giad058)
Supplement: giad058_Reviewer_2_Report_Original_Submission — CÃ©sar BernabÃ© -- 4/15/2023 Reviewed [file giad058_reviewer_2_report_original_submission.pdf]

Reviewer Report

Title: Lessons learned to boost a bioinformatics knowledge base reusability, the Bgee experience

Version: Original Submission **Date:** 4/15/2023

Reviewer name: CÃ©sar BernabÃ©

Reviewer Comments to Author:

The paper "Boosting interoperability: towards an increasingly reusable bioinformatics knowledge base" provides detailed information about use cases implemented by the Bgee to enhance its interoperability with other databases. The use cases described are important as they provide knowledge to the community and help other interested parties find solutions to similar challenges. The paper is well-written, falls within the scope of the journal, and makes a valuable contribution to the literature. However, certain aspects of the paper could benefit from greater clarification.

In the introduction, it is mentioned that ontologies only "elevate heterogeneity problems at a higher level". Although I agree with this statement, I understand that the use of ontologies can also result in fewer heterogeneity problems (i.e., heterogeneity occurrences). Specifically, I argue that the number of ontologies that define overlapping domains or concepts is significantly smaller than the number of datasets that collects information about the same domains or concepts. I think the authors share the same thought, but the way these observations are presented in the paper creates room for different expectations. When I first read the sentence above, I expected that the authors would discuss possible solutions to the problem of ontology heterogeneity throughout the paper, which did not happen.

I like the distinction between different types of interoperability (one-side, two-side and multi-side). However, I feel that the relationship between the types and the use cases is not clearly presented, and I have some difficulty understanding which type of interoperability approach is used in each case. I suggest including a summary table with the cases, and the interoperability approaches used in each one (and maybe other important points?).

About one-side and multi-side interoperability: I wonder how to classify a case where one side strictly complies with the other side (one-sided?), but the other side implements a third-party standard (multi-sided?). The true question behind this is: are the definitions of interoperability types clear enough? It seems to me that there are cases in which more than one classification is possible.

Finally, I have a few questions regarding the use cases. In general, it is unclear to me why a particular approach is used in each case, whether other approaches are possible, and what the pros and cons are. For example, why exchanging CSV files in some approaches and implementing a federated query in others? Are these choices made because they are the only viable option? Disclosing how the authors came up with these recommendations would help readers to identify the best approach for their use cases. Overall, the paper would benefit from adding a discussion section that contrasts and compares the use cases.

Methods

Are the methods appropriate to the aims of the study, are they well described, and are necessary controls included? Choose an item.

Conclusions

Are the conclusions adequately supported by the data shown? Choose an item.

Reporting Standards

Does the manuscript adhere to the journal's guidelines on minimum standards of reporting? Choose an item.

Choose an item.

Statistics

Are you able to assess all statistics in the manuscript, including the appropriateness of statistical tests used? Choose an item.

Quality of Written English

Please indicate the quality of language in the manuscript: Choose an item.

Declaration of Competing Interests

Please complete a declaration of competing interests, considering the following questions:

- Have you in the past five years received reimbursements, fees, funding, or salary from an organisation that may in any way gain or lose financially from the publication of this manuscript, either now or in the future?
- Do you hold any stocks or shares in an organisation that may in any way gain or lose financially from the publication of this manuscript, either now or in the future?
- Do you hold or are you currently applying for any patents relating to the content of the manuscript?
- Have you received reimbursements, fees, funding, or salary from an organization that holds or has applied for patents relating to the content of the manuscript?
- Do you have any other financial competing interests?
- Do you have any non-financial competing interests in relation to this paper?

If you can answer no to all of the above, write 'I declare that I have no competing interests' below. If your reply is yes to any, please give details below.

I declare that I have no competing interests.

I agree to the open peer review policy of the journal. I understand that my name will be included on my report to the authors and, if the manuscript is accepted for publication, my named report including any attachments I upload will be posted on the website along with the authors' responses. I agree for my

report to be made available under an Open Access Creative Commons CC-BY license (http://creativecommons.org/licenses/by/4.0/). I understand that any comments which I do not wish to be included in my named report can be included as confidential comments to the editors, which will not be published.

Choose an item.

To further support our reviewers, we have joined with Publons, where you can gain additional credit to further highlight your hard work (see: https://publons.com/journal/530/gigascience). On publication of this paper, your review will be automatically added to Publons, you can then choose whether or not to claim your Publons credit. I understand this statement.

Yes Choose an item.
