# Supplementary material for: Lessons learned to boost a bioinformatics knowledge base reusability, the Bgee experience
Source: Gigascience. 2023 Aug 17;12:giad058. doi: 10.1093/gigascience/giad058 (PMC10433096; doi:10.1093/gigascience/giad058)
Supplement: giad058_Reviewer_2_Report_Revision_1 — CÃ©sar BernabÃ© -- 6/16/2023 Reviewed [file giad058_reviewer_2_report_revision_1.pdf]

Reviewer Report

Title: Lessons learned to boost a bioinformatics knowledge base reusability, the Bgee experience

Version: Revision 1 **Date:** 6/16/2023

Reviewer name: CÃ©sar BernabÃ©

Reviewer Comments to Author:

The reviewed version of the paper is improved in terms of structure, readability, and motivation. The new title is clearer, and most suggestions from the previous review round have been addressed. Congratulations on the good work.
I agree with reviewer #1 that the first version of the paper seemed more like a discussion of the potential of Bgee. Although the new proposed title and the additions to the main text partially address this issue, I am not convinced by the changes in the abstract. When the authors say "The findings are driven by several real-world scenario examples that were mostly implemented by Bgee, a well-established gene expression database, and other major knowledge bases", it still gives the same impression as the first version of the paper. Overall, I feel that the abstract has not kept up with the improvements in the rest of the paper. For example, I suggest that the abstract should say something similar to the last paragraph of the introduction: "To further demonstrate they are transferable, for each Bgee interoperability experience, we will also highlight, when it is applicable, similar implementations by widely used bioinformatics KBs such as GeneCards (a human gene-centric KB), UniProtKB (a protein-centric KB) and Orthologous MAtrix (OMA, an orthology resource)."
In the highlighted reusability sentence in the first paragraph of the introduction: are you suggesting a new definition of reusability for your own work or reusing one from literature? If the latter, please include a reference (NB: the definition of interoperability is referenced).
I also wonder about the claims made in the second half of the second paragraph of the Introduction. I partially disagree that the principles should not be dissociated nor seen as independent. A FAIRification project should indeed have findability, accessibility, interoperability and reusability as its ultimate goal. However, when the letters are broken down into the 15 principles, the dependency claim no longer holds. This is because some principles are more closely related to some than others, and because some should be prioritised differently depending on the FAIRification objectives. For example, A1.1 and A1.2 are more dependent on each other than on F1. Also, some principles (e.g., F2) can be achieved in different manners depending on the domain where they are being applied. Finally, there must be room for some independence in order for the FAIR implementation to be viable (it is infeasible to implement all the principles at once).
Secondly, I do not think that publications 3 and 4 lead to the conclusion that the structure and abstraction of the principles in four different letters has been the source of confusion and misinterpretation by researchers. Some may get the impression that these claims come from such publications (e.g. as a motivation for writing these papers), which is not true. I acknowledge the problem of confusion, but the authors also fail to mention that these principles have been made "abstract" (I would say agnostic) to respect the particularities of each research community. That is, the principles do

not dictate an exact implementation, as this should be defined by each community according to its own needs.
I have also had second thoughts about the example given in the third paragraph of the introduction. I think it is possible to be findable or accessible without being interoperable (in fact, this assertion contradicts the authors' claim that the four letters cause confusion). The authors seem to be collapsing different technical/semantic layers to which FAIR can be related. In other words, there are different levels of interoperability to be addressed, e.g. technical (the example given is the authors) and semantic (on the data and metadata). A counterexample would be a data resource that is findable through its metadata (because the metadata could be interoperated/managed by different systems (e.g. the server and the indexer/search engine)). However, the data that the metadata points to is not interoperable at all. I understand that this statement is based on the authors' own interpretation of interoperability (as given at the beginning of the same paragraph). This claim should be accompanied by a statement that "F" and "A" are not possible without "I", according to the authors' understanding of Interoperability. Alternatively, a more appropriate fix would be to change the motivation (e.g., "I" is one of the most targeted aspects of FAIR).
The end of the last paragraph in the introduction section is slightly disconnected. In fact, this paragraph is very long and would benefit from some reorganisation. In the second part of the paragraph (i.e., "Furthermore, …"): are the authors citing related works? Are they also mentioned as similar implementations? I suggest elaborating this part as another paragraph focused on related works (or even considering making it a subsection).
I appreciate the improvements in clarifying the interoperability approaches and I think Table 1 has improved readability. However, I am not sure that Figure 1 provides any additional information compared to Table 1 and Figure 2. The technologies used are not linked to the knowledge base with which they were used, which makes the information a bit unclear. I suggest removing Figure 1 and adding the technologies used (e.g. RDF, Java API, Python API, etc.) as an extra column in Table 1 or making them all explicit in Figure 2.
Figure 2 is very informative and summarises the work well. I suggest adding some extra information/semantics to the relationships (the arrows). For instance, how should the arrow between "Generate Bgee data exchange files" and "EasyBgee SQL dump" be interpreted? Is it also an import? Or is it a "create" action?
About Fig 11: I wonder how informative/complex it can be for the average reader. Should it be replaced by an infographic or a more didactic figure? Readers with a technical/related background could refer to a GitHub reference to the XML, which should be included in the text.
For the sake of better organisation of the information/text, I suggest that "Final implementation considerations" becomes a subsection (i.e., 4.4).

Methods

Are the methods appropriate to the aims of the study, are they well described, and are necessary controls included? Choose an item.

Conclusions

Are the conclusions adequately supported by the data shown? Choose an item.

Reporting Standards

Does the manuscript adhere to the journal's guidelines on minimum standards of reporting? Choose an item.

Choose an item.

Statistics

Are you able to assess all statistics in the manuscript, including the appropriateness of statistical tests used? Choose an item.

Quality of Written English

Please indicate the quality of language in the manuscript: Choose an item.

Declaration of Competing Interests

Please complete a declaration of competing interests, considering the following questions:

- Have you in the past five years received reimbursements, fees, funding, or salary from an organisation that may in any way gain or lose financially from the publication of this manuscript, either now or in the future?
- Do you hold any stocks or shares in an organisation that may in any way gain or lose financially from the publication of this manuscript, either now or in the future?
- Do you hold or are you currently applying for any patents relating to the content of the manuscript?
- Have you received reimbursements, fees, funding, or salary from an organization that holds or has applied for patents relating to the content of the manuscript?
- Do you have any other financial competing interests?
- Do you have any non-financial competing interests in relation to this paper?

If you can answer no to all of the above, write 'I declare that I have no competing interests' below. If your reply is yes to any, please give details below.

I declare that I have no competing interests

I agree to the open peer review policy of the journal. I understand that my name will be included on my report to the authors and, if the manuscript is accepted for publication, my named report including any attachments I upload will be posted on the website along with the authors' responses. I agree for my report to be made available under an Open Access Creative Commons CC-BY license (http://creativecommons.org/licenses/by/4.0/). I understand that any comments which I do not wish to

be included in my named report can be included as confidential comments to the editors, which will not be published.

Choose an item.

To further support our reviewers, we have joined with Publons, where you can gain additional credit to further highlight your hard work (see: https://publons.com/journal/530/gigascience). On publication of this paper, your review will be automatically added to Publons, you can then choose whether or not to claim your Publons credit. I understand this statement.

Yes Choose an item.
