# Supplementary material for: Lessons learned to boost a bioinformatics knowledge base reusability, the Bgee experience
Source: Gigascience. 2023 Aug 17;12:giad058. doi: 10.1093/gigascience/giad058 (PMC10433096; doi:10.1093/gigascience/giad058)
Supplement: giad058_Reviewer_2_Report_Revision_2 — CÃ©sar BernabÃ© -- 6/30/2023 Reviewed [file giad058_reviewer_2_report_revision_2.pdf]

Reviewer Report

Title: Lessons learned to boost a bioinformatics knowledge base reusability, the Bgee experience

Version: Revision 2 **Date:** 6/30/2023

Reviewer name: CÃ©sar BernabÃ©

Reviewer Comments to Author:

Dear authors,
I appreciate your effort in addressing some of the suggestions I made in the prior round of reviews. I acknowledge that some of my comments may have led to additional confusion and I made an effort to better articulate my suggestions throughout the remainder of the text.
Before I discuss the authors' responses, I would like to clarify my approach to scientific work. First, I recognise and appreciate the diverse perspectives held by individuals on various scientific subjects. The multiplicity of viewpoints contributes to the fascinating nature of scientific exploration, and I do not think that disagreements should be seen as a misunderstanding from any of the sides. Moreover, if a reviewer "misunderstands" a particular sentence, then I would see this as an invitation to improve the clarity of this sentence.
Second, I want to point out that I mentioned some suggestions in the second round of reviews, not in the first round, because I felt that the manuscript first required major changes to effectively communicate its main message. In the second round, I pointed towards more detailed issues because the authors managed to substantially improve the paper in the first round, and I applaud the authors for the first round of revisions.
Finally, I understand that the review process should be a mutually beneficial task. The friendlier this process, the more beneficial it is for everyone involved. With that in mind, I made some suggestions that, in my opinion, would improve the quality of the paper. I am completely fine with the authors' decision to disregard some of the suggestions. However, there are areas of major concern that require careful revision, before I can provide a final recommendation to the editor.
I acknowledge that discussing FAIR is out of the scope of the paper. Nevertheless, the paper makes certain claims about the FAIR principles, and those must be addressed:
1. About my argument regarding the principles being seen as dissociated ones: I understand the authors' view on the (dis)association of the FAIR letters. My point was that F, A, I and R should not be seen as dissociated for resources aimed to be FAIR (e.g., a resource that is "F" and "R" is not considered "FAIR" - all letters are needed). However, while the letters should not be dissociated, the fifteen principles (e.g., F1, F2, A1..) and their implementation (here I mean implementation as a process) should be dealt with with certain independence. This is not contradictory to the comment made by Reviewer #1, but complementary. I invite the authors to revisit this section and check their claims one more time.
2. In the prior round of reviews, I pointed out that references [3] and [4] do not support the claim that the four different letters have been the source of confusion and misinterpretation by researchers. The authors responded by stating that these references "highlight our opinion that later publications were needed in order to better understand the FAIR principles". I find it questionable to cite literature in

order to make a stronger point about one's opinion. I suggest rewriting this section and make it clear that you are stating your own opinion.
3. Finally, let me come back to the example given in the third paragraph. I did not mean to say that the authors are having second interpretations or thoughts. I apologise if I conveyed this impression. Instead, I meant to say that your example makes sense according to the definition of interoperability used in this paper. However, this definition is not the only interoperability definition given in the literature. As mentioned in my previous response, there are different layers in which interoperability can be perceived, and there are different interoperability definitions for each layer. I suggest making it clear that the given example holds true in the context of the technical definition of interoperability that you are using. You could, for example, add an explanatory footnote.
I hope that the authors perceive my recommendations as valuable feedback that will help them to improve the quality of the paper.
All the best.

Methods

Are the methods appropriate to the aims of the study, are they well described, and are necessary controls included? Choose an item.

Conclusions

Are the conclusions adequately supported by the data shown? Choose an item.

Reporting Standards

Does the manuscript adhere to the journal's guidelines on minimum standards of reporting? Choose an item.

Choose an item.

Statistics

Are you able to assess all statistics in the manuscript, including the appropriateness of statistical tests used? Choose an item.

Quality of Written English

Please indicate the quality of language in the manuscript: Choose an item.

Declaration of Competing Interests

Please complete a declaration of competing interests, considering the following questions:

- Have you in the past five years received reimbursements, fees, funding, or salary from an organisation that may in any way gain or lose financially from the publication of this manuscript, either now or in the future?
- Do you hold any stocks or shares in an organisation that may in any way gain or lose financially from the publication of this manuscript, either now or in the future?
- Do you hold or are you currently applying for any patents relating to the content of the manuscript?
- Have you received reimbursements, fees, funding, or salary from an organization that holds or has applied for patents relating to the content of the manuscript?
- Do you have any other financial competing interests?
- Do you have any non-financial competing interests in relation to this paper?

If you can answer no to all of the above, write 'I declare that I have no competing interests' below. If your reply is yes to any, please give details below.

I declare that I have no competing interests.

I agree to the open peer review policy of the journal. I understand that my name will be included on my report to the authors and, if the manuscript is accepted for publication, my named report including any attachments I upload will be posted on the website along with the authors' responses. I agree for my report to be made available under an Open Access Creative Commons CC-BY license (http://creativecommons.org/licenses/by/4.0/). I understand that any comments which I do not wish to be included in my named report can be included as confidential comments to the editors, which will not be published.

Choose an item.

To further support our reviewers, we have joined with Publons, where you can gain additional credit to further highlight your hard work (see: https://publons.com/journal/530/gigascience). On publication of this paper, your review will be automatically added to Publons, you can then choose whether or not to claim your Publons credit. I understand this statement.

Yes Choose an item.
